# Virological and Immunological Outcomes of an Intensified Four-Drug versus a Standard Three-Drug Antiretroviral Regimen, Both Integrase Strand Transfer Inhibitor-Based, in Primary HIV Infection

**DOI:** 10.3390/ph15040403

**Published:** 2022-03-26

**Authors:** Annalisa Mondi, Carmela Pinnetti, Patrizia Lorenzini, Maria Maddalena Plazzi, Isabella Abbate, Marta Camici, Chiara Agrati, Elisabetta Grilli, Francesca Gili, Rozenn Esvan, Nicoletta Orchi, Gabriella Rozera, Alessandra Amendola, Federica Forbici, Caterina Gori, Roberta Gagliardini, Rita Bellagamba, Adriana Ammassari, Stefania Cicalini, Maria Rosaria Capobianchi, Andrea Antinori

**Affiliations:** 1 HIV/AIDS Department, National Institute for Infectious Diseases, Lazzaro Spallanzani IRCCS, 00149 Rome, Italy; carmela.pinnetti@inmi.it (C.P.); patrizia.lorenzini@inmi.it (P.L.); maria.plazzi@inmi.it (M.M.P.); marta.camici@inmi.it (M.C.); elisabetta.grilli@inmi.it (E.G.); roberta.gagliardini@inmi.it (R.G.); rita.bellagamba@inmi.it (R.B.); adriana.ammassari@inmi.it (A.A.); stefania.cicalini@inmi.it (S.C.); andrea.antinori@inmi.it (A.A.); 2 Laboratory of Virology, National Institute for Infectious Diseases, Lazzaro Spallanzani IRCCS, 00149 Rome, Italy; isabella.abbate@inmi.it (I.A.); gabriella.rozera@inmi.it (G.R.); alessandra.amendola@inmi.it (A.A.); federica.forbici@inmi.it (F.F.); caterina.gori@inmi.it (C.G.); maria.capobianchi@inmi.it (M.R.C.); 3 Cellular Immunology and Pharmacology Laboratory, National Institute for Infectious Diseases, Lazzaro Spallanzani IRCCS, 00149 Rome, Italy; chiara.agrati@inmi.it; 4 AIDS Regional Referral Center, National Institute for Infectious Diseases, Lazzaro Spallanzani IRCCS, 00149 Rome, Italy; francesca.gili@inmi.it (F.G.); rozenn.esvan@inmi.it (R.E.); nicoletta.orchi@inmi.it (N.O.)

**Keywords:** primary HIV infection, antiretroviral therapy, integrase stand transfer inhibitors, rapid ART

## Abstract

The optimal therapeutic approach for primary HIV infection (PHI) is still debated. We aimed to compare the viroimmunological response to a four- versus a three-drug regimen, both INSTI-based, in patients with PHI. This was a monocentric, prospective, observational study including all patients diagnosed with PHI from December 2014 to April 2018. Antiretroviral therapy (ART) was started, before genotype resistance test results, with tenofovir/emtricitabine and either raltegravir plus boosted darunavir or dolutegravir. Cumulative probability of virological suppression [VS] (HIV-1 RNA< 40 cp/mL), low-level HIV-1 DNA [LL-HIVDNA] (HIV-1 DNA < 200 copies/10^6^PBMC), and CD4/CD8 ratio ≥1 were estimated using Kaplan–Meier curves. Factors associated with the achievement of VS, LL-HIVDNA, and CD4/CD8 ≥ 1 were assessed by a Cox regression model. We enrolled 144 patients (95.8% male, median age 34 years): 110 (76%) started a four-drug-based therapy, and 34 (24%) a three-drug regimen. Both treatment groups showed a comparable high probability of achieving VS and a similar probability of reaching LL-HIVDNA and a CD4/CD8 ratio ≥1 after 48 weeks from ART initiation. Higher baseline HIV-1 RNA and HIV-1 DNA levels lowered the chance of VS, whereas a better preserved immunocompetence increased that chance. Not statistically significant factors associated with LL-HIVDNA achievement were found, whereas a higher baseline CD4/CD8 ratio predicted the achievement of immune recovery. In PHI patients, the rapid initiation of either an intensified four-drug or a standard three-drug INSTI-based regimen showed comparable responses in terms of VS, viral reservoir size, and immunological recovery.

## 1. Introduction

Current HIV treatment guidelines recommend starting a long-life antiretroviral therapy (ART) during primary HIV infection (PHI) [1,2]. This indication is supported by the evidence of the multiple benefits resulting from the initiation of ART during this stage of infection, including the preservation of the immunological function [3,4], the containment of the viral reservoirs size [5,6], and, through a rapid virological suppression, the limitation of the viral mutation rate [7] and the decrease of infection transmission risk [8]. However, the achievement of these benefits seemed to be closely related to the time of ART initiation, with a greater effect in the earlier stages of HIV infection [5,9,10]. Thus, in the last years, in the setting of PHI, an increasing attention has been focused on rapid ART initiation strategies which have demonstrated high acceptability and excellent efficacy [11,12].

One of most relevant benefits of starting ART during PHI is the achievement of lower levels of immune activation during ART-mediated viral suppression [13], which have been associated with an increased risk of all-cause of morbidity and mortality [14]. Indeed, the very early phases of HIV infection are characterized by high levels of pro-inflammatory cytokines and markers which decline during the first several months of ART, gradually achieving a stable “setpoint” [13]. The initiation of ART during acute HIV infection has been demonstrated to significantly reduce the inflammatory response. However, the levels of several immune-activation markers remained persistently elevated, even in patients starting the therapy within the first 2–3 weeks from infection [15], suggesting an extremely early HIV-induced damage to the immune system and a persistent higher risk for non-AIDS related outcomes also in patients treated with a rapid ART approach during PHI.

Despite the progresses achieved in the management of PHI, the best treatment strategy for this phase of HIV infection is still debated, and direct comparisons between different therapeutic approaches are still limited [16,17,18,19,20]. The current guidelines do not frankly differentiate the treatment options for acute and chronic HIV infection, only recommending the use of regimens with higher genetic barrier, such as those containing boosted protease inhibitors (PI/b) or second-generation integrase strand transfer inhibitors (INSTIs), to allow an immediate therapy initiation before genotyping resistance testing (GRT) results [1,2].

INSTIs might represent key drugs to be first administered during PHI thanks to their good tolerability, the more rapid decay of viremia they induce compared to other antiretrovirals, and the high concentration they achieve in genital secretions [21]. In this setting, INSTIs, particularly raltegravir (RAL), were initially evaluated as part of four- or five-drug intensified regimens but failed to demonstrate significant advantages in terms of mid- and long-term virological and immunological responses compared to standard ART [16,17,18,22]. More recently, INSTIs-based triple regimens administered as initial therapy for PHI have shown good efficacy and tolerability [23,24], with faster viral suppression and immune recovery compared to PI/b regimens [19,20]. However, direct comparisons between INSTI-based intensified regimens and INSTI triple ART are lacking.

The aim of this study was to evaluate and compare the efficacy and feasibility of two INSTI-based treatment strategies in patients starting ART during PHI: a RAL-intensified four-drug regimen and a dolutegravir (DTG)-based triple regimen.

## 2. Results

### 2.1. Baseline Characteristics and Follow-Up

A total of 144 patients were included in the study and followed for a median observation time of 21 months (IQR 8.4–34). The main baseline characteristics of the study population are shown in Table 1. Briefly, almost all patients (95.8%) were male, with a median age of 34 years (IQR 27–43), and most of them acquired HIV through homosexual intercourses (81.9%). CD4 cell count was above 500 cell/mm^3^ in 84 (58.3%) patients, whereas 35 (25%) subjects had a CD4/CD8 cell ratio ≥ 1. Median HIV-1 RNA and HIV-1 DNA at baseline were 5.6 log10 copies/mL (IQR 4.8–6.6) and 4.4 log10 copies/10^6^ PBMC (IQR 3.8–4.8), respectively. Fiebig stages at baseline were II/III in 22 (15.3%), IV in 45 (31.2%), V in 41 (28.5%), and VI in 33 (22.9%) participants. Overall, ART was started within a median time of 5 days (IQR 3–9) from HIV diagnosis, with an intensified four-drug regimen in 110 (76.4%) patients and with a three-drug regimen in 34 (23.6%) patients. Specifically, 123 patients (85.4%) started ART within 14 days from HIV diagnosis, of whom 102 in the first 7 days. At baseline, the four-drug and three-drug ART groups did not differ for the main characteristics except for the time from HIV diagnosis to ART initiation, which was slightly shorter for the former (*p* = 0.021).

Over the study period, linkage to care was 93% (10 patients were lost to follow-up: 3 changed the referral center, 6 of the remaining 7 patients returned to clinical observation within approximately 2 years).

The results of GRTs, available for 140 of the 144 patients, showed that mutations conferring resistance to NNRTI (K103N, G190A, V106A, E138A) were detected in 12/140 (8.7%) patients, whereas mutations conferring resistance to NRTI (M41L, M184V) were found in 2/140 (1.4%) subjects. Although neither major PI nor INSTI mutations were detected, two patients, both in the intensified ART arm, harbored a virus with the T97A accessory resistance mutations, conferring potential low-level resistance to first-generation INSTI, according to the Stanford HIV drug resistance database (score 10). Only for one of these latter patients the initial ART was modified (Supplementary Table S1).

### 2.2. Virological Outcomes

**Figure 1:**
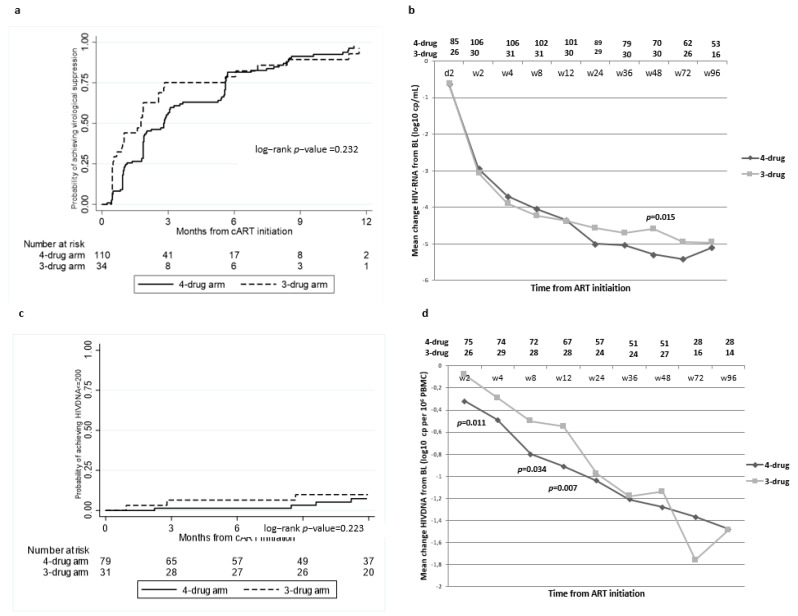
Probabilities of achieving virological suppression (*n* = 144 patients) (**a**) and low-level HIV-DNA (*n* = 110 patients.) * (**c**) according to the treatment group. Evolution of HIV-RNA (**b**) and HIV-DNA (**d**) at different time points according to the treatment **. * Patients with baseline HIVDNA ≥ 200 available and at least a following HIVDNA assessment. ** Not significant *p*-values in the comparison between the four-drug and the three-drug arms are not shown.

Overall, during the follow-up, 130 subjects (90.3%) achieved virological suppression. The estimated probabilities of achieving an HIV-1 RNA below 40 copies/mL after 12, 24, and 48 weeks of therapy were 56% (95% confidence interval, CI 47.3–66.2), 82% (95% CI 73.2–88.6), and 98% (95% CI 92.3–99.5%) for patients starting the four-drug ART and 75% (95%, CI 59.7–88.2), 79% (95% CI 63.4–90.1), and 96% (95% CI 84.8–99.7) for patients starting the three-drug regimen, without statistically significant differences between the two treatment groups (*p* = 0.232) (Figure 1a). The mean decay of HIV-1 RNA levels over 96 weeks was comparable between the two arms, except for a lower reduction in viral load for the four-drug ART arm at week 48 from treatment start [*p* = 0.015] (Figure 1b). When exploring factors associated with virological suppression at the adjusted Cox regression model, higher baseline levels of HIV-1 RNA and HIV-1 DNA were associated with a lower chance of achieving virological suppression (adjusted hazard ratio [aHR] 0.68 per each HIV-1 RNA log higher, *p* = 0.007; aHR 0.56 per each HIV-1 DNA log higher, *p* = 0.026). Conversely, a more preserved immune competence at treatment start (aHR 1.97 for CD4 cell count > 500 cells/mm^3^ versus ≤500 cells/mm^3^, *p* = 0.009) and having acquired HIV through heterosexual compared to homosexual intercourses (aHR 2.14, *p* = 0.033) favorably predicted the achievement of virological suppression (Table 2). These findings were also confirmed in the OT analysis, censoring those patients who switched the first antiretroviral regimen (*n* = 17). Over the follow-up, five patients experienced virological failure (four in the four-drug group and one in the three-drug group), with an incidence rate of 2.5% (95% CI 1.0–5.9) without differences between the treatment groups (*p* = 0.526).

Data about HIV-1 DNA were available for a subgroup of 110 patients. Over the observation time, 10 (9.1%) subjects achieved low-level HIV-1 DNA. The cumulative probabilities of reaching this outcome in the four-drug and three-drug arms were 1.4 (95%CI 0.2–9.3) and 6.5% (95%CI 1.7–23.4), respectively, at 12 and 24 weeks, and 7.4% (95%CI 2.8–18.7) and 9.9% (95%CI 3.3–27.7), respectively, at 48 weeks, without significant differences between the two groups (*p* = 0.223) (Figure 1c). When considering the slope of HIV-1 DNA decay, the mean decrease of HIV-1 DNA appeared to be significantly more pronounced in the intensified treatment group than in the standard ART group over the first 3 months (four-drug arm versus three-drug arm at week 2 −0.32 versus −0.08, *p* = 0.011; at week 8: −0.8 versus −0.5, *p* = 0.034; at week 12: −0.91 versus −0.55, *p* = 0.07), while afterwards it was comparable (Figure 1d). At multivariable analysis, although none of the analyzed factors were significantly associated with low-level HIV-DNA achievement, both a higher level of HIV-1 DNA at baseline and a later start of the therapy after HIV diagnosis showed a borderline significance in the association, with a decreased chance of low viral reservoir achievement (aHR= 0.30 per each HIV-1 DNA log higher, *p* = 0.052; aHR = 0.09 for patients starting ART at Fiebig stage IV versus stages I/II, *p* = 0.058) (Table 3).

### 2.3. Immunological Outcomes

**Figure 2:**
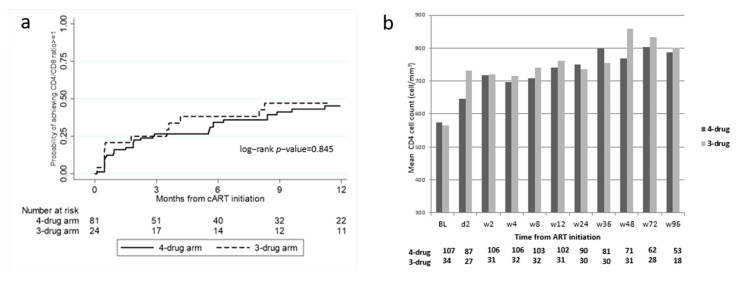
Probabilities of achieving a CD4/CD8 ratio ≥ 1 (*n* = 105 patients) * (**a**) and evolution of CD4 cell count ** (**b**) at different time points according to the treatment. * Patients with baseline CD4/CD8 available and <1. ** Not significant *p*-values in the comparison between the four-drug and the three-drug arms are not shown.

The mean CD4 cell count gradually improved at each time point, with a similar mean increase between the groups (Figure 2b). Of 105 patients with baseline CD4/CD8 ratio available and below 1, 48 (45.7%) patients achieved a CD4/CD8 ratio ≥ 1. The estimated probabilities of reaching a CD4/CD8 ratio ≥ 1 in the four-drug ART and the three-drug ART groups were 27% (95%CI 18.2–37.8) and 25% (95%CI 12.1–47.4) at week 12, 34% (95%CI 24.7–46.3) and 38% (22.0–60.7) at week 24, and 45% (95% CI 34.1–58.1) and 47% (29.4–68.9) at week 48, respectively, without differences between the groups (log-rank test, *p* = 0.845) (Figure 2a). At multivariable analysis, only a higher CD4/CD8 ratio at ART initiation was significantly associated with the achievement of a CD4/CD8 ratio ≥ 1 (aHR 2.96 CD4/CD8 ratio 0.78–0.99 versus <0.44, *p* = 0.011) (Table 4).

### 2.4. Adherence

No differences in self-reported adherence between the two treatment groups were found at week 24 (*p* = 0.408) and week 48 of ART (*p* = 0.443).

## 3. Discussion

In this observational study, including patients diagnosed with PHI from December 2014 to April 2018, both an intensified four-drug regimen based on RAL and DRV/b and a DTG-based triple ART, showed a comparable virological efficacy with a very high probability of achieving HIV-1 RNA suppression (98% versus 96% at week 48, *p* = 0.232) and a similar chance of reaching low-level HIV-1 DNA (<200 copies per 10^6^PBMC), which was lower than 10% after 48 weeks of ART (7.4% versus 9.9%, *p* = 0.223).

Our findings are in line with those of several clinical trials which failed to demonstrate significant advantages in terms of both virological suppression and size of viral reservoirs in patients starting intensified four- or five-drug antiretroviral regimens compared to those treated with standard PI- or NNRTI-based triple ART [16,17,18,22,25]. On the contrary, a recent study found that starting ART with regimens not recommended by the current guidelines, including those intensified with more than three drugs, increased the risk of an incomplete viral response in patients diagnosed with acute or early HIV infection [26].

Of note, in contrast with previous trials comparing standard three- versus four- or five-drug regimens [16,18,22], we did not observe a different decay of plasma viral load between the standard and the intensified treatment arms. A possible explanation of this result is that in our cohort, unlike in the above-mentioned studies, RAL-intensified ART was compared to an INSTI-based regimen which is characterized by a more rapid decrease of viral load compared to PI/b or NNRTI-based regimens [27]. As evidence of this, in a previous study comparing a RAL-based triple ART with the same regimen intensified with MVC, the viral load decreased rapidly in both treatment groups but slightly slower in the intensified-ART arm [25].

Although INSTIs have been recommended as anchor drugs of choice for chronic HIV infections for nearly a decade, data on their use in the context of PHI have been accumulating only in more recent years. A large French study on a cohort of patients with PHI documented an earlier virological suppression and a faster immune restoration in patients starting INSTI-based versus boosted-PI-based regimens. These results were confirmed after restricting the analysis to patients starting DTG versus boosted DRV as third drug [19]. The achievement of a faster viral load suppression with INSTI- (mainly RAL) versus boosted-PI (mainly DRV)-based regimens was confirmed in a smaller cohort of patients with early HIV infection [20].

Additionally, the role of another INSTI, elvitegravir/cobicistat/emtricitabine/tenofovir disoproxil fumarate (EVG/c/TDF/FTC), in the setting of PHI, was explored in two small studies which showed a rapid and sustained virological suppression, a significant immunological recovery, and an optimal safety of this regimen [23,24]. Nevertheless, the use of EVG/C/TDF/FTC in PHI has been questioned, owing to the lower genetic barrier of EVG compared to DTG or bictegravir, which can lead to a potential higher risk of virological failure in the presence of polymorphic substitutions and accessory mutations of the integrase gene conferring low-level resistance to first-generation INSTIs [28]. Despite the role of these substitutions in determining virological failure in naïve patients being unknown and their reported prevalence in PHI ranging widely from 1.5% to 13.9% [24,29], the use of INSTIs with higher genetic barrier in the context of PHI, characterized by very high viral loads and the need of a prompt ART start, seems reasonable. To this regard, a recent Italian study reported a 13.3% prevalence of INSTIs polymorphisms or substitutions in a small cohort of patients diagnosed with early or recent HIV infection between January 2015 and June 2016 [30]. In contrast to these data, in our cohort, only two patients (1.4%) harbored INSTI accessory resistance mutations (both T79A). Moreover, although most patients started ART within 14 days and, particularly, within the first week from HIV diagnosis, before the availability of GRT, only one patient needed to change therapy according to the GRT results (presence of T79A INSTI polymorphism).

When exploring predictive factors, as expected, the virological burden as well as the immunological competence at baseline positively predicted virological suppression. The achievement of low-level HIV-1 DNA seemed to be associated with pre-therapeutic HIV-1 DNA levels and the timing of ART initiation. Although these associations did not reach statistically significance, these findings are consistent with previous studies reporting a correlation between the size of viral reservoir and both the pre-treatment HIV-1 DNA level [29,31] and the timing of ART initiation [5,32]. It is worth noting that, in this study, the type of antiretroviral regimen used was not associated with any of the virological outcomes.

The immune recovery was comparable between the intensified- and the standard-ART arms. Notably, the probability of achieving a CD4/CD8 ratio ≥1 within the first year of ART initiation approached 50% in both groups (45% in the four-drug arm versus 47% in the three-drug arm, *p* = 0.845). This rapid normalization of the CD4/CD8 ratio, already observed in the setting of PHI [33], is in contrast with data from chronic HIV infection indicating that a similar rate of normalization was achieved after a median of 10 years with suppressive ART [34]. Although we did not confirm a significant association between the timing of ART initiation and immune recovery, as already reported [9,33], the finding that a more preserved immunological condition at the time of therapy start strongly predicted the achievement of a CD4/CD8 ratio ≥1 might suggest a key role of an early ART in immune restoration

Finally, our study confirmed the optimal results of a rapid ART approach in PHI patients in terms of linkage to care (more than 90%) and acceptability, as already demonstrated in previous reports [12,20].

This study has some limitations, including the limited size of the population, examined, particularly in the three-drug arm, its observational nature, which is prone to bias due to unmeasured confounders, and the short follow-up.

## 4. Materials and Methods

### 4.1. Study Population

This is a retrospective analysis of prospectively collected data from the SIREA (observational cohort study of HIV-1-infected patients with Acute Retroviral Syndrome) cohort. SIREA is a monocentric cohort study set up in 2014, enrolling patients, aged at least 18 years, diagnosed with PHI at the National Institute for Infectious Diseases “Lazzaro Spallanzani” in Rome, Italy. Demographic and epidemiological data and symptoms at HIV diagnosis were collected and recorded for all participants in an anonymous form at baseline. Viro-immunological and therapeutic data along with blood specimens were collected at baseline and, afterward, at each time point, as reported below. All consecutive patients enrolled in the SIREA cohort from December 2014 to April 2018 who started ART with tenofovir/emtricitabine (TDF/FTC) associated with either boosted darunavir (DRV/b) and RAL or DTG were included in the present analysis.

### 4.2. Virological Assessment

HIV serodiagnosis was performed with the fourth-generation HIV-1/2 Architect HIV antigen (Ag)/antibody (Ab) Combo assay, Abbott (Illinois Park, IL, USA). Reactive serum samples underwent confirmation with HIV-1 Western Blot (WB) (New Lav I Bio-Rad, Hercules, CA, USA) using World Health Organization (WHO) criteria (two env products reactivity). An avidity test was carried out, on confirmed positive samples lacking p31 reactivity, to discriminate between recent (<6 months) and late infections. PHI was diagnosed and staged using the Fiebig system, which categorized the infections on the basis of the following criteria: (1) positive HIV viremia (at least 2000 copies/mL) with negative fourth-generation HIV assay (Ab/Ag Combo) (Fiebig I); (2) positive HIV Ab/Ag Combo test and negative (Fiebig II/III) or undetermined WB test (Fiebig IV); (3) positive HIV Ab/Ag Combo test and incomplete WB test (no p31 protein reactivity, Fiebig V); (4) positive HIV-1 enzyme immunoassay (EIA) with a complete WB test but a documented negative HIV-1 EIA within the previous 6 months or with an avidity test <0.8 (Fiebig VI) [35,36]. HIV-1 RNA was measured using the Abbott Real-Time HIV-1 assay (Abbott Molecular, Inc., Des Plaines, IL, USA, 40 copies/mL lower limit detection). Total HIV-1 DNA was extracted from peripheral blood mononuclear cells (PBMCs) by the QIAsymphony DNA Midi Kit (QIAGEN, S.r.l. Milan, Italy) and quantified by real-time polymerase chain reaction (PCR) targeting the long terminal repeat (LTR) region. DNA was amplified with the sense primer NEC 152 (GCCTCAATAAAGCTTGCCTTGA) and the reverse primer NEC 131 (GGCGCCACTGCTAGAGATTTT) in the presence of a dually (FAM and TAMRA) labelled NEC LTR probe (AAGTAGTGTGTGCCCGTCTGTTRTKTGACT). For the standard curve, dilutions of 8E5 cell DNA containing 1 proviral copy per cell were used. An additional real-time PCR targeting the housekeeping cellular hTERT gene was used to refer total HIV-1 DNA copies to one million PBMC [37].

### 4.3. Antiretroviral Treatments and Timing of the Evaluations

At enrollment, the subjects underwent a complete clinical and viroimmunological evaluation, including GRT. ART was started, generally before the GRT results, with one of the following regimens: (a) 4-drug ART consisting of TDF/FTC 245/200 mg plus RAL 400 mg twice daily (BID) plus either DRV/ritonavir (DRV/r) 800/100 mg or, from November 2016, DRV/cobicistat (DRV/c) 800/150 mg or (b) 3-drug ART with TDF/FTC 245/200 mg plus DTG 50 mg once daily (QD) from May 2015. Virological and immunological data (HIV-RNA, HIV-DNA, lymphocyte T cells count) were collected at baseline (defined as the date of ART initiation), on day 2 (except for HIV-DNA), at week 2, week 4, week 8, week 12, week 24, week 36, and week 48 and thereafter every six months. Adherence was evaluated through a self-reported visual analogic scale (VAS) ranging from 1 to 100, at the same time points, except for baseline and day 2.

### 4.4. Outcomes

The primary outcome was to estimate and compare the probability of virological suppression between patients on the 4-drug regimen versus patients on the 3-drug regimen. Secondary outcomes were to evaluate and compare the probabilities of the groups of reaching low-level HIV-1 DNA and a CD4/CD8 ratio ≥1, the trend of HIV-1 RNA and HIV-1 DNA decay, the evolution of CD4 cell count, and the self-reported adherence. Additionally, factors associated with the achievement of the main virological and immunological outcomes were assessed.

### 4.5. Definitions

Virological suppression was defined as the first achievement of HIV-1 RNA < 40 copies/mL. Virological failure was considered as either incomplete suppression (a viral load >200 copies/mL after 6 months of ART) or viral rebound (two consecutive HIV-1 RNA > 50 cp/mL or an HIV-1 RNA > 1000 cp/mL after the achievement of virological suppression). In this study, the amount of HIV-1 DNA defining a low-level HIV-1 DNA was set at <200 copies/106 PBMC based on the ANRS 116 SALTO study in which this threshold was associated with a lower probability to reassume therapy after ART interruption [38]. The same threshold of HIV-1 DNA has been already used in the setting of ART started during both chronic and acute HIV infection to define, together with immunological parameters, the status of optimal viroimmunological responders [32]. This value was also reached by the 25th percentile of subjects who started ART during acute HIV infection after 24 months of treatment [39]. Suboptimal adherence was defined as VAS < 100. Finally, the transmitted drug resistance mutations were determined according to the WHO recommended surveillance drug resistance mutation list, updated in 2009, for PIs, nucleoside transcriptase reverse inhibitors (NRTIs), NNRTIs [40], integrated with the Stanford HIV drug resistance database for INSTIs [41].

### 4.6. Statistical Analysis

Categorical variables are described as absolute numbers and percentage frequencies; continuous variables are quoted as medians and interquartile range (IQR). The baseline characteristics of the two groups were compared using Student’s *t*-test or Mann–Whitney U-test for continuous variables, as appropriate, or Chi-square test for categorical variables.

Kaplan–Meier curves were used to estimate the probability of primary and secondary outcomes, and log-rank test was used to compare the survival distributions of the two groups. Factors associated with virological suppression, CD4/CD8 ratio ≥1, and low-level HIV-1 DNA achievement were evaluated by a multivariable Cox proportional hazard model. The covariates included in the models were chosen a priori as potentially associated with the outcomes. The adherence, measured by visual analogic scale (VAS) at various time points, was included in multivariable models as a time-updated covariate. Mean changes from baseline of HIV-1 RNA and HIV-1 DNA levels as well as CD4 cell count at each time point were assessed and compared between the two treatment arms using the Student’s *t* test. Self-reported adherence was assessed by VAS at baseline and at weeks 24 and 48. The mean adherence VAS scores at weeks 24 and 48 were compared between the two groups using the Student’s *t*-test

An intention-to-treat (ITT) approach, ignoring switches to first-line therapy, was adopted for all the analysis. Additionally, an on-treatment (OT) approach, censoring patients who switched to first-line therapy, was applied for the primary outcome. A two-tailed *p*-value < 0.05 was considered to be statistically significant. All analyses were performed using STATA 15.1 software.

## 5. Conclusions

In this study, we provide further evidence that in PHI patients, an intensified four-drug regimen including RAL and DRV/b and a DTG-based standard regimen showed comparable responses in terms of virological suppression, viral reservoir size, and immunological recovery. These data support the use of second-generation INSTI-based triple regimens as first-line ART in this setting, also in order to limit cost and pill burden. Moreover, our findings confirmed that a rapid ART approach in this stage of infection is related to a high acceptability and viro-immunological response.

## Figures and Tables

**Table 1:** Main baseline characteristics of the total population and according to the treatment.

	4-Drug ARM (TDF/FTC + DRV/b + RAL) *n* = 110	3-Drug ARM (TDF/FTC + DTG) *n* = 34	*p*	Total Population *n* = 144
**Male gender, *n* (%)**	107 (97.2)	31 (91.2)	0.120	138 (95.8)
**Age years, median (IQR)**	34 (26–45)	35 (28–39)	0.832	34 (27–43)
**Mode of HIV transmission, *n* (%)**			0.065	
Homosexual contact	95 (86.4)	23 (67.7)		118 (81.9)
Heterosexual contact	13 (11.8)	10 (29.4)		23 (16.0)
IDU	1 (0.9)	1 (2.9)		2 (1.4)
Other/Unknown	1 (0.9)	-		1 (0.7)
**Non-Italian born, *n* (%)**	11 (10.0)	6 (17.7)	0.235	17 (11.8)
**Days from HIV diagnosis to ART,**				
**median (IQR)**	5 (2–7)	6 (4–17)	**0.021**	5 (3–9)
***n* (%)**				
≤7	83 (75.5)	19 (55.9)	**0.050**	102 (70.8)
8–14	15 (13.6)	6 (17.6)		21 (14.6)
≥15	12 (10.9)	9 (26.5)		21 (14.6)
**BL CD4 count, cells/μL, median (IQR)**	557 (379–686)	564 (383–729)	0.946	557 (383–697)
**BL CD4 count, cells/μL, *n* (%)**			0.516	
≤500 cell/μL	41 (37.3)	16 (47.1)		57 (39.6)
>500 cell/μL	66 (60.0)	18 (52.9)		84 (58.3)
Missing	3 (2.7)	-		3 (2.1)
**BL CD4/CD8 ratio ≥ 1, *n* (%)**	25 (23.6)	10 (29.4)	0.495	35 (25.0)
**BL HIV-RNA, log_10_ cp/mL, median (IQR)**	5.7 (5.0–6.5)	5.5 (4.4–6.6)	0.503	5.6 (4.8–6.6)
**BL HIV-RNA, *n* (%)**				
HIVRNA ≤ 500.000 cp/mL	54 (49.1)	14 (41.2)	0.524	68 (47.2)
HIVRNA > 500.000 cp/mL	55 (50.0)	19 (55.9)		74 (51.4)
Missing	1 (0.9)	1 (2.9)		2 (1.4)
**BL HIV-DNA, log_10_ cp/10^6^PBMC, median (IQR)**	4.6 (3.8–4.9)	4.1 (3.8–4.7)	0.228	4.4 (3.8–4.8)
**BL Fiebig stage, *n* (%)**			0.172	
II/III	17 (15.4)	5 (14.7)		22 (15.3)
IV	35 (31.8)	10 (29.4)		45 (31.2)
V	35 (31.8)	6 (17.7)		41 (28.5)
VI	20 (18.2)	13 (38.2)		33 (22.9)
missing	3 (2.7)	-		3 (2.1)
**Boosted PI in the regimen, *n* (%)**			-	
DRV/r	72 (65.5)	-		72 (50.0)
DRV/c	38 (34.5)	-		38 (26.4)
**Observation time in months, median (IQR)**	19 (8–35)	23 (17–29)	0.839	21 (8.4–34)

Notes: Abbreviations: TDF = tenofovir disoproxil fumarate; FTC = emtricitabine; DRV/b = darunavir/ritonavir or darunavir/cobicistat; RAL = raltegravir; DTG = dolutegravir; IQR = interquartile range; IDU = Intravenous Drug User; ART = antiretroviral therapy; BL = baseline; PBMC = peripheral blood mononuclear cell; Cp = copies; PI = protease inhibitor; DRV/r = darunavir/ritonavir; DRV/c = darunavir/cobicistat. Bold values represent statistically significant *p*-values.

**Table 2:** Multivariable Cox model fit: relative hazards of achieving virological suppression.

	Univariate		Multivariate	
	HR (95% CI)	*p*-Value	aRH (95% CI)	*p*-Value
**Age**				
per 10 years older	0.91 (0.77–1.07)	0.260		
**Mode of HIV transmission**				
Homosexual	1.00		1.00	
Heterosexual	1.49 (0.94–2.36)	0.093	2.14 (1.06–4.29)	**0.033**
IDU	1.71 (0.23–12.45)	0.597	-	-
**Baseline CD4 count, cells/mm^3^**				
>500 vs. ≤500	2.08 (1.43–3.02)	**<0.001**	1.97 (1.18–3.29)	**0.009**
**Baseline CD4/CD8 ratio**				
≥1 vs. <1	1.76 (1.18–2.64)	**0.006**	1.45 (0.72–2.92)	0.299
**Baseline HIV-RNA**				
per 1 log higher	0.47 (0.38–0.57)	**<0.001**	0.68 (0.51–0.90)	**0.007**
**Baseline HIV-DNA**				
per 1 log higher	0.49 (0.35–0.68)	**<0.001**	0.56 (0.34–0.93)	**0.026**
**Baseline Fiebig stage**				
II/III	1.00			
IV	0.82 (0.47–1.41)	0.465		
V	0.82 (0.48–1.42)	0.487		
VI	1.27 (0.72–2.23)	0.411		
**ART regimen**				
3-drug vs. 4-drug arm	1.27 (0.85–1.91)	0.244	1.34 (0.76–2.37)	0.318
**Adherence**				
VAS < 100 (time-updated)	0.87 (0.52–1.45)	0.597	1.32 (0.71–2.46)	0.377

Notes: Abbreviations: HR, unadjusted hazard ratio; aHR, adjusted hazard ratio; CI, confidence intervals; IDU, intravenous drug users; ART, antiretroviral therapy; vs, versus; VAS, visual analogic scale. Bold values represent statistically significant *p*-values.

**Table 3:** Multivariable Cox model fit: relative hazards of achieving HIVDNA < 200 copies/10^6^PBMC (*n* = 110 patients ^).

	Univariate		Multivariate	
	HR (95% CI)	*p*-Value	aRH (95% CI)	*p*-Value
**Baseline CD4 count, cells/mm^3^**				
>500 vs. ≤500	1.46 (0.38–5.64)	0.584		
**Baseline CD4/CD8 ratio**				
≥1 vs. <1	1.46 (0.38–5.65)	0.582		
**Baseline HIV-RNA**				
per 1 log higher	0.44 (0.26–0.74)	**0.002**	0.62 (0.30–1.31)	0.210
**Baseline HIV-DNA**				
per 1 log higher	0.20 (0.08–0.54)	**0.001**	0.30 (0.09–1.01)	0.052
**Baseline Fiebig stage**				
II/III	1.00		1.00	
IV	0.11 (0.01–1.26)	0.076	0.09 (0.01–1.09)	0.058
V	-	-	-	-
VI	1.21 (0.25–5.81)	0.814	0.23 (0.02–2.18)	0.201
**ART regimen**				
3-drug vs. 4-drug arm	2.56 (0.74–8.84)	0.137	1.92 (0.51–7.17)	0.333

Notes: Abbreviations: HR, unadjusted hazard ratio; aHR, adjusted hazard ratio; CI, confidence intervals; ART, antiretroviral therapy; vs., versus; Bold values represent statistically significant *p*-values ^ Patients with baseline HIVDNA ≥ 200 available and at least a following HIVDNA assessment.

**Table 4:** Multivariable Cox model fit: relative hazards of achieving CD4/CD8 > 1 (*n* = 105 ^).

	Univariate		Multivariate	
	HR (95% CI)	*p*-Value	aRH (95% CI)	*p*-Value
***Days from HIV diagnosis to ART start***				
*≤7*	1.00	-	1.00	-
*8–14*	1.51 (0.66–3.48)	0.328	1.19 (0.46–3.04)	0.718
*≥15*	1.94 (1.00–3.74)	**0.049**	1.42 (0.68–2.98)	0.349
***Baseline CD4 count, cells/mm^3^***				
*>500 vs. ≤500*	2.48 (1.34–4.58)	**0.004**	1.73 (0.89–3.38)	0.108
***Baseline CD4/CD8 ratio (by quartiles)***				
*<0.44 (Q1)*	1.00	-	1.00	-
*0.45–0.77 (Q1–Q2)*	2.02 (0.94–4.33)	**0.007**	2.08 (0.88–4.94)	**0.095**
*0.78–0.99 (Q2–Q3)*	2.70 (1.29–5.68)	**0.009**	2.96 (1.29–6.79)	**0.011**
***Baseline HIV-RNA***				
*per 1 log higher*	0.83 (0.64–1.08)	0.164	1.06 (0.75–1.49)	0.750
***Baseline HIV-DNA***				
*per 1 log higher*	0.65 (0.42–1.01)	0.053	0.74 (0.45–1.21)	0.233
***ART regimen***				
3-drug vs. 4-drug arm	1.06 (0.55–2.04)	0.856	0.82 (0.41–1.64)	0.571

Notes: Abbreviations: HR, unadjusted hazard ratio; aHR, adjusted hazard ratio; CI, confidence intervals; ART, antiretroviral therapy; vs., versus; Q = quartile. Bold values represent statistically significant *p*-values. ^ Patients with baseline CD4/CD 8 ratio available and <1.

## Data Availability

Data is contained within the article and supplementary material.

## Supplementary Materials

The following supporting information can be downloaded at: https://www.mdpi.com/article/10.3390/ph15040403/s1, Table S1: Description of patients with major resistance mutations and INSTIs polymorphisms/substitutions at the pre-ART GRT: viroimmunological data and therapeutic management.
