# Supplementary material for: Virological and Immunological Outcomes of an Intensified Four-Drug versus a Standard Three-Drug Antiretroviral Regimen, Both Integrase Strand Transfer Inhibitor-Based, in Primary HIV Infection
Source: Pharmaceuticals (Basel). 2022 Mar 26;15(4):403. doi: 10.3390/ph15040403 (PMC9024471; doi:10.3390/ph15040403)
Supplement: Supplementary file 1 [file pharmaceuticals-15-00403-s001.zip › pharmaceuticals-1642886-supplementary.pdf]

Table S1: Description of patients with major resistance mutations and INSTIs polymorphisms/substitutions at the pre-ART GRT: viroimmunological data and therapeutic management.

	NNRTIs, NRTIs and PIs Major Resistance Mutations/INSTIs Polymorphism or Substitutions	Baseline VL (copies/mL)	Baseline CD4 Count (cell/mm^3)	Initial ART	ART Change	Reason for Changing
**Patient 1**	**K103N**	3.873.430	354	TDF/FTC+DRV/r+RAL	Y	simplification
**Patient 2**	**E138A**	6.018	951	TDF/FTC+DRV/r+RAL	Y	simplification
**Patient 3**	**G190A**	225.709	605	TDF/FTC+DRV/r+RAL	Y	simplification
**Patient 4**	**M41L; V106A**	183.894	343	TDF/FTC+DRV/r+RAL	Y	simplification
**Patient 5**	**E138A**	108.779.000	341	TDF/FTC+DRV/r+RAL	Y	simplification
**Patient 6**	**K103N**	10.733	876	TDF/FTC+DTG	N	/
**Patient 7**	**E138A**	18.474.400	427	TDF/FTC+DTG	N	/
**Patient 8**	**K103N**	44612	359	TDF/FTC+DTG	N	/
**Patient 9**	**E138G**	332.394	697	TDF/FTC+DTG	N	/
**Patient 10**	**E138A**	165398	319	TDF/FTC+DRV/r+RAL	Y	simplification
**Patient 11**	**M184V**	160929	529	TDF/FTC+DRV/r+RAL	Y	simplification
**Patient 12**	**E138A**	44463	429	TDF/FTC+DRV/C+RAL	N	/
**Patient 13**	**E138A**	345518	155	TDF/FTC+DRV/C+RAL	N	/
**Patient 14**	***T79A***	38732	988	TDF/FTC+DRV/R+RAL	**NA**	
**Patient 15**	***T79A***	7378	625	TDF/FTC+DRV/C+RAL	**Y**	RAL withdrawal due to T97A

Notes: Abbreviations: NNRTIs, non-nucleoside reverse transcriptase inhibitors; NRTIs, nucleoside reverse transcriptase inhibitors PIs protease inhibitors; INSTI integrase stand transfer inhibitors, VL viral load, ART antiretroviral regimen, TDF tenofovir disoproxil fumarate, FTC emtricitabine, DTG dolutegravir, RAL raltegravir, DRV/r darunavir/ritonavir, DRV/c darunavir/cobicistat, N no, Y yes.
